# Immunogenicity Assessment of Different Segments and Domains of Group a Streptococcal C5a Peptidase and Their Application Potential as Carrier Protein for Glycoconjugate Vaccine Development

**DOI:** 10.3390/vaccines9020139

**Published:** 2021-02-09

**Authors:** Guirong Wang, Jielin Zhao, Yisheng Zhao, Subo Wang, Shaojie Feng, Guofeng Gu

**Affiliations:** National Glycoengineering Research Center and Shandong Key Laboratory of Carbohydrate Chemistry and Glycobiology, Shandong University, 72 Binhai Road, Qingdao 266237, China; wanggr@mail.sdu.edu.cn (G.W.); zjl8212175@163.com (J.Z.); shrinelibra@gmail.com (Y.Z.); wang_subo@163.com (S.W.); fengsj1989@126.com (S.F.)

**Keywords:** group A streptococcus, group A streptococcal C5a peptidase, subunit protein vaccine, immunogenicity, carrier protein

## Abstract

Group A streptococcal C5a peptidase (ScpA) is a highly conserved surface virulence factor present on group A streptococcus (GAS) cell surfaces. It has attracted much more attention as a promising antigenic target for GAS vaccine development due to its high antigenicity to stimulate specific and immunoprotective antibodies. In this study, a series of segments of ScpA were rationally designed according to the functional domains described in its crystal structure, efficiently prepared and immunologically evaluated so as to assess their potential as antigens for the development of subunit vaccines. Immunological studies revealed that Fn, Fn2, and rsScpA193 proteins were promising antigen candidates worthy for further exploration. In addition, the potential of Fn and Fn2 as carrier proteins to formulate effective glycoconjugate vaccine was also investigated.

## 1. Introduction

The Gram-positive bacterium *Streptococcus pyogenes*, also known as group A streptococcus (GAS), is a uniquely human pathogen that is responsible for a variety of fatal poststreptococcal sequelae, such as rheumatic fever, rheumatic heart disease, and acute glomerulonephritis [1,2,3]. These fatal infections are usually associated with high morbidity and mortality in human, causing at least 500,000 deaths and more than 700 million prevalent cases annually [4,5]. Over last decades, many highly conserved virulence factors that exposed on the cell surface of GAS, such as M protein [6,7,8,9,10], group A carbohydrate (GAC) [11,12,13,14,15,16,17], C5a peptidase [18,19], and interleukin-8 cleaving peptidase (SpyCEP) [20,21,22,23], etc., have been studied as potential antigenic targets for GAS vaccine development. Till now, however, no commercial human GAS vaccines are available, making the development of safe and effective vaccines against GAS infections an urgent subject [24,25,26,27,28].

Group A streptococcal C5a peptidase (ScpA) is a cell surface endopeptidase that specifically inactivates human phagocyte C5a chemotaxin via proteolytic cleavage at its leukocyte-binding site of the amino acid residues His67 and Lys68 [29,30]. This can affect lymphocyte recruitment and retard bacterial clearance from the host [31]. Studies have revealed that ScpA is highly conserved expressed among almost all serotypes of GAS [32]. It is also highly immunogenic and could potentially provide efficacious immunoprotection against streptococcal infection [19,33,34]. For example, intranasal immunization of mice with the active-site deletion form of ScpA could significantly provoke high levels of specific IgA and IgG antibodies that efficiently protected the nasopharyngeal colonization of different GAS serotypes [33]. Likewise, subcutaneous immunization of mice with the active-site mutation form of ScpA also stimulated protective immune responses which accelerated clearance of GAS as compared to control mice inoculated with tetanus toxoid (TT) [18]. Thus, ScpA has been an attractive antigenic target for GAS vaccine development. Further studies in children have indicated that ScpA was highly immunogenic in human and the elicited antibodies could neutralize the activity of natural ScpA [34]. More recently, ScpA has been also used as one antigenic component for combination vaccines, whose immunities were well identified in a nonhuman primate model or a humanized plasminogen AlbPLG1 mice model [35,36,37,38]. In our previous study, we demonstrated that an enzymatically inactive ScpA mutant (His193Ala mutation, ScpA193) provoked robust T cell-mediated immune responses featured by the production of high titers of ScpA-specific IgG antibodies [39].

**Figure 1:**
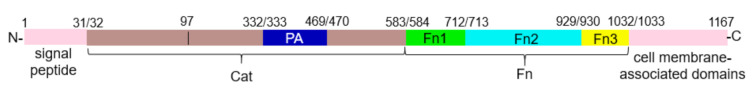
Schematic representation of the domain organization of ScpA. Cat domain is salmon, PA domain is blue, three Fn domains are green, cyan, and yellow, respectively, and the signal peptide and cell membrane-associated domains are pink. Numbers indicate domain boundaries in ScpA.

Structurally, ScpA is a multi-domain protein composed of 1167 amino acid residues in length and possesses an N-terminal 31-residue signal peptide, a catalytic domain (residues 32–583, Cat domain) with an inserted protease-associated subdomain (residues 333-469, PA domain), three tandemly arranged fibronectin domains (residues 584–712, Fn1; residues 713–929, Fn2; residues 930–1032, Fn3), and several cell membrane-associated domains (residues 1033–1167) at the C-terminus (Figure 1) [40]. Most ScpA proteins currently studied for GAS vaccine development are nearly full-length mutant forms [18,39]. To the best of our knowledge, however, immunological properties of the different domains of ScpA have not been reported yet. Thus, it is extraordinarily deserved to explore their immunogenicity so as to identify the more antigenic epitope for subunit protein vaccine development. In this study, we recombined the different segmented domains of ScpA and evaluated the detail immunological properties of these recombinant proteins in murine model.

## 2. Materials and Methods

### 2.1. Expression and Purification of Protein Antigens

Expression vector pGEX-6P-3 carrying the *ScpA* gene (bases 96–3096) that encoded amino acid residues 32–1032 of *S. pyogenes* B220 was provided as a gift by Professor Jakki C. Cooney from the University of Limerick (Ireland), and the expression vector pGEX-6P-3 carrying the mutational *ScpA193* gene with the histidine-193 residue replaced by the alanine residue was previously constructed from our laboratory [39]. Using the designed primers listed in Table S1, the DNA sequences of *rsScpA*, *rsScpA193*, *Cat*, *Fn*, *PA*, *Fn1*, *Fn2* and *Fn3* genes were acquired from two vectors mentioned above and thus amplified. All amplified DNA fragments were constructed in plasmid pGEX-6P-3 (for *rsScpA*, *rsScpA193*, *Fn*, *PA*, *Fn1*, *Fn2* and *Fn3* genes) or plasmid pET-21b (for *Cat* gene). PCR reactions were performed to obtain the amplified fragments and transformed into *Escherichia coli* DH5a. Thereafter, these extracted recombinant plasmids were then transferred into *Escherichia coli* BL21(DE3) competent cell and the resultant *E. coli* strain BL21(DE3) was grown in the LB medium including 100 μg mL^−1^ of antibiotic ampicillin and induced with 0.75 mM of isopropyl 1-β-D-thiogalactoside (IPTG) at 16 °C for 24 h. Then, all cells were collected and lysed via sonication (VCX800, SONICS, Newtown, CT, USA) on ice. rsScpA, rsScpA193, Fn, PA, Fn1, Fn2 and Fn3 proteins were purified by the GST-Sepharose FF column, while Cat protein was purified by the Ni^+^ affinity column. The recombinant GST-tag proteins were incubated with Prescission Protease to remove the GST tags and purified by GST-Sepharose FF again, and the purified proteins were stored at −80 °C.

### 2.2. Evaluation of the Enzymatic Activities of rsScpA, rsScpA193 and Cat Proteins

The human C5a peptide substrate (0.6 μg) was dissolved in 50 mM Tris–HCl buffer (pH 7.5, 20 μL) containing 100 mM of NaCl and 5 mM of CaCl_2_, and different amounts of each recombinant protein (0.02, 0.06, 0.6, 6.0 μg) were added. After incubating at 20 °C for 30 min, 1 μL of the reaction mixture was spotted onto a stainless-steel MALDI-TOF MS plate and then covered with sinapinic acid matrix. The sample plate was dried and then subjected to MS analysis with a MALDI-TOF mass spectrometer (AXIMA Confidence, Shimadzu Co., Manchester, UK) with linear-positive mode ionization.

### 2.3. Preparation of the GAC Trisaccharide–Protein Conjugates

A mixture of GAC trisaccharide (3 mg) and di-(*N*-succinimidyl)-glutarate (DSG, 15 equiv, 26 mg) in co-solvents of DMF and PBS buffer (0.1 M, pH 8.0) (*v*/*v* 4:1, 0.5 mL) was gently stirred at rt for 4 h, and the solvents were then removed under reduced pressure. The activated monoester product was precipitated with nine volumes of ethyl acetate and the excess DSG was removed by completely washing the resultant precipitate with ethyl acetate (×10). The white solid was then dried under high vacuum and used directly for protein conjugation. Thereafter, a solution of the activated trisaccharyl monoester and Fn, Fn2, rsScpA193 or CRM197 (oligosaccharide/protein, 1:2 mass ratio) in PBS buffer (0.1 M, 0.5 mL, pH 8.2) was stirred at rt for 3 days. After no further increase in molecular mass that monitored with MALDI-TOF MS analysis (sinapinic acid matrix) had occurred, the reaction was dialyzed against distilled water (3 × 4 mL) using an Amicon ultrafiltration cell equipped with a Diaflo membrane (Ultracel, 10 kDa). Fractions that contained glycoproteins were combined and lyophilized to give the corresponding protein-trisaccharide conjugates (3.82 mg for Fn-Tri; 5.52 mg for Fn2-Tri; 5.40 mg for rsScpA193-Tri and 4.22 mg for CRM197-Tri) as white powders. The carbohydrate loading of each glycoconjugate was determined by means of MALDI-TOF MS and calculated according to the following Equation (1):

Carbohydrate loading% = (MS of conjugate -MS of protein)/MS of conjugate × 100%(1)

### 2.4. Immunization of Mice

Each protein antigen (200 μg of rsScpA193, Cat, Fn, PA, Fn1, Fn2 or Fn3), or each glycoconjugate (0.5~1.5 mg, containing 30 μg of trisaccharide per Fn-Tri, Fn2-Tri, rsScpA193-Tri or CRM197-Tri) were dissolved in 0.5 mL of 2×PBS buffer, and then mixed with 0.5 mL of Freund’s complete adjuvant (FCA, F5881, Sigma, St. Louis, MO, USA)/Freund’s incomplete adjuvant (FIA, F5506, Sigma) to prepare an emulsion. Each group of six female BALB/c mice (6–8 weeks old) was subcutaneously injected with 0.1 mL of the CFA emulsion on day 1 and boosted three times with 0.1 mL of the IFA emulsion on days 15, 22 and 29, respectively. Blood samples were collected via the tail vein of each mouse on day 0 before and on day 35 and day 56 after the immunization. The antisera were obtained from the collected blood samples by the standard protocols and then stored at −80 °C before immunological analysis. All care and handling of animals in this study were performed in strict accordance with the National Institute for Health Guide for the Care and Use of Laboratory Animals (National Research Council, 8th Ed, National Academies Press (US): Washington, DC., 2011), and approved by the Institutional Animal Care and Use Committee at Shandong University.

### 2.5. Enzyme-Linked Immunosorbent Assay (ELISA)

A solution of corresponding coating antigen (100 μL/well, 2 μg/mL) dissolved in coating buffer (0.1 M aq bicarbonate, pH 9.6) were incubated on ELISA plates at 4 °C overnight and then at 37 °C for 1 h. Then, the plates were washed with PBS buffer containing 0.05% Tween-20 (PBST) three times and incubated with blocking buffer (1% BSA in PBST) at rt for 1 h, followed by washing with PBST three times again. Thereafter, the serial dilutions from 1:300 to 1:1968300 of each mouse serum in PBS (100 μL/well) were added to the coated plates followed by incubation at 37 °C for 2 h. After the coated plates were washed with PBST three time, a 1:1000 diluted solution of alkaline phosphatase-linked goat anti-mouse total (anti-kappa), IgM, IgG, IgG1, IgG2a, IgG2b or IgG3 (ab99631, ab98672, ab98710, ab98690, ab98695, ab98700 and ab98705, Abcam, Cambridge, UK) antibodies were added to the plates (100 μL/well) and incubated at rt for 1 h. After being washed with PBST three times, the plates were treated with a *p*-nitrophenyl phosphate (PNPP) solution (1.67 mg/mL in buffer, 100 μL) for 30 min at rt, and then quenched by addition of quenching solution (3 M NaOH, 25 μL) to each well. The plates were read at 405 nm wavelength using a microplate reader. After deducting the background optical density (OD) values of day 0 sera, the OD_405_ values against serum dilution values were plotted to obtain a best-fit equation line. The antibody titer was calculated at the inverse of the dilution value at which an OD value of 0.1 was achieved.

### 2.6. Flow Cytometry (FCM) Analysis

GAS J17A4 (ATCC 12385) and group B streptococci (GBS, also known as *Streptococcus agalactiae*, ATCC BAA-1138) bacteria were cultured in brain heart infusion medium (BHIM, 237500, BD Biosciences, Franklin Lakes, NJ, USA) and harvested by centrifugation at 12,000× *g* for 10 min. After being washed twice with PBS, cells (1 × 10^7^ CFU) were resuspended in 100 μL of PBS buffer, and then incubated with 2 μL of day 0 or day 56 antiserum at 4 °C for 1 h. The treated cells were washed twice with PBS again and then incubated with fluorescein isothiocyanate (FITC)-linked goat anti-mouse IgG antibody (1 μL in 100 μL PBS) at 4 °C for 1 h. Finally, the cells were washed and suspended in 1 mL of PBS and then subjected to FCM analysis using an ACEA NovoCyte instrument (san Diego, CA, USA).

## 3. Results and Discussion

To utilize ScpA protein as an antigen candidate, it is necessary to prepare its enzymatically inactivated form to guarantee biosafety. The N-terminal catalytic triad residues of Asp130, His193 and Ser512 in the Cat domain enable the inherent enzymatic activity of ScpA [41,42]. It has been well documented that mutagenesis of one or two of these residues, especially His193 residue, in the active sites of ScpA could obviously decrease its enzymatic activity [39,41,42]. Furthermore, it was reported that the peptide (Asn32-Asp79/Lys90) embodied in the Cat domain could be autoproteolytically degraded at Ala71, Asp79 or Lys90 residue site during protein maturation [42]. Based on the aforementioned findings, we planned to choose a truncated form of mature ScpA protein sequence encoding amino acid residues 97-1032 for systematic investigation. Accordingly, four small functional segments, PA, Fn1, Fn2 and Fn3, and two functional proteins, Cat (residues 97–583) and Fn (residues 584–1032), of ScpA were designed and explored their possibility as subunit vaccines. In addition, the full-size mature ScpA protein (residues 97–1032, rsScpA) and its enzymatically inactive mutant (His193Ala, rsScpA193) were also designed, respectively, as positive controls to be used in the following enzymatic assay and immunological study.

### 3.1. Preparation of Recombinant Target Proteins

**Figure 2:**
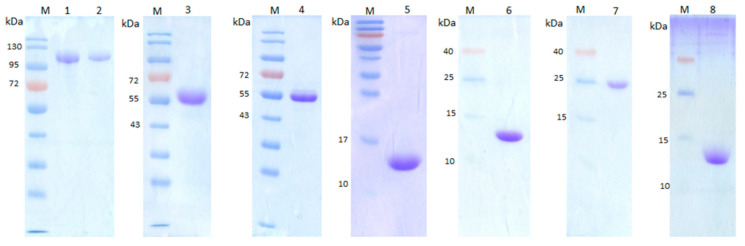
SDS-PAGE analysis of purified recombinant proteins. Lane 1: rsScpA, Lane 2: rsScpA193, Lane 3: Cat, Lane 4: Fn, Lane 5: PA, Lane 6: Fn1, Lane 7: Fn2, Lane 8: Fn3. M: molecular maker.

Using the primers listed in Table S1, *rsScpA*, *rsScpA193*, *Cat*, *PA*, *Fn*, *Fn1*, *Fn2* and *Fn3* genes were successfully cloned from the previously reported pGEX-6P-3 expression vectors that carried *ScpA* and *ScpA193* genes (bases 96–3096) via conventional protocols [39,40]. After these genes were inserted into expression vector pGEX-6P-3 (for *rsScpA*, *rsScpA193*, *PA*, *Fn*, *Fn1*, *Fn2* and *Fn3* genes) and pET-21b (for *Cat* gene) by standard protocols, the recombinant plasmids were transferred into *E. coli* BL21(DE3) to express the corresponding proteins. The recombinant proteins were designed to contain a GST tag at the N-terminal or a hexa-histidine tag at the C-terminal. Therefore, rsScpA, rsScpA193, PA, Fn, Fn1, Fn2 and Fn3 proteins with GST tags were purified by GSTrap affinity column, while Cat protein, which contained a hexa-histidine tag, was purified by Ni^+^ affinity column. The SDS-PAGE results depicted in Figure 2 showed the purity and homogeneity of the purified recombinant target proteins. The detected bands of rsScpA (~103 kDa), rsScpA193 (~103 kDa), Cat (~52.6 kDa), Fn (~50.3 kDa), PA (~15 kDa), Fn1 (~14.6 kDa), Fn2 (~24.1 kDa) and Fn3 (~11.3 kDa) were consistent with the theoretically predicted molecular weights.

**Figure 3:**
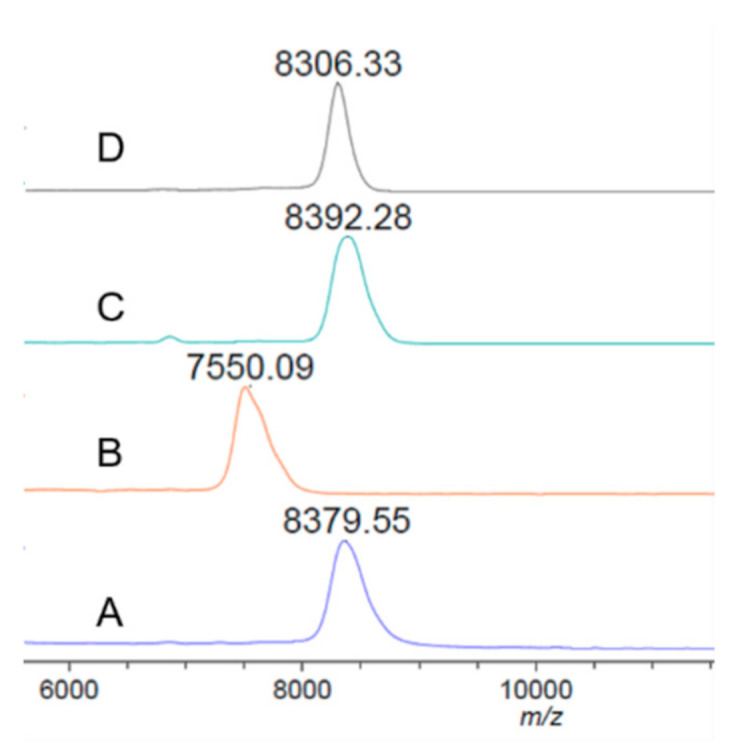
MALDI-TOF MS results for the enzymatic hydrolysis of (**A**) human C5a peptide (30 μg/mL) catalyzed by (**B**) rsScpA (1.0 μg/mL), (**C**) rsScpA193 (300 μg/mL) and (**D**) Cat (300 μg/mL) after incubation at 20 °C for 30 min.

The enzymatic activities of recombinant rsScpA193 and Cat proteins were assayed with a simple and sensitive MALDI-TOF MS-based detection method [39]. The commercial recombinant human C5a peptide was used as substrate for enzymatic reaction. The reactions were carried out under similar reaction conditions as previously reported protocol [39]. After incubation of substrate C5a peptide (30 μg/mL) with different concentrations of each tested protein (1.0~300 μg/mL) at 20 °C for 30 min, the reaction mixtures were directly subjected to MALDI-TOF MS analysis. As shown in Figure 3, the wild-type rsScpA showed an excellent enzymatic activity that could completely transform C5a peptide (Figure 3A, ~8380 Da) into the product (Figure 3B, ~7550 Da) at a concentration of 1.0 μg/mL. On the contrary, both rsScpA193 and Cat proteins exhibited no enzymatic activities toward substrate C5a peptide even at a concentration of 300 μg/mL (Figure 3C,D). These results indicated rsScpA193 and Cat proteins were inactive and could be used as potential antigens for immunological evaluation in this study.

### 3.2. Immunological Evaluation of Segmented Domains of ScpA Protein

With enough amounts of homogeneous recombinant rsScpA193, Cat, Fn, PA, Fn1, Fn2 and Fn3 proteins in hand, we carried out systematic evaluation and comparison of their immunological characteristics. Accordingly, BALB/c mice were subcutaneously inoculated with each protein (20 μg per inoculation per mouse) emulsified in Freund adjuvant (FCA for initial immunization and FIA for boosting immunizations) on days 1, 15, 22 and 29, respectively. The corresponding antisera were prepared by clotting blood samples that collected on day 0 before initial immunization and day 35 and day 56 after boosting immunization, and then subject to ELISA to determine specific total (anti-kappa), IgM, IgG antibodies, as well as IgG subtypes including IgG1, IgG2a, IgG2b and IgG3 antibodies, using corresponding proteins as the capture antigens.

**Figure 4:**
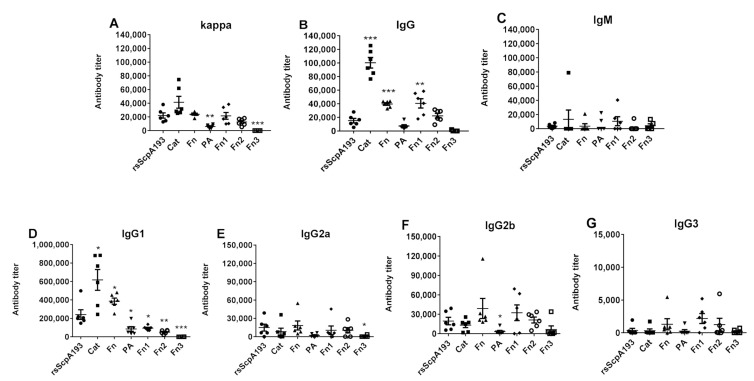
ELISA results of the corresponding protein-specific (**A**) total (anti-kappa), (**B**) total IgG, (**C**) IgM, (**D**) IgG1, (**E**) IgG2a, (**F**) IgG2b, and (**G**) Ig3 antibody titers in the pooled day 35 antisera are displayed. Each dot represents the result of an individual mouse immunized with rsScpA193 (●), Cat (■), Fn (▲), PA (▼), Fn1 (♦), Fn2 (○), and Fn3 (□), respectively; the black bars show the mean ± SEM values. *: significantly different (*p* < 0.05); **: very significantly different (*p* < 0.01); ***: extremely significantly different (*p* < 0.001) from the data of antiserum rsScpA193.

As shown in Figure 4, except for Fn3 protein that hardly induced significant immune responses, all other segment proteins elicited high titers of anti-kappa and IgG antibodies (Figure 4A,B), as well as very low titers of IgM antibodies (Figure 4C) in the pooled day 35 antisera. The production of higher titers of total IgG antibodies indicated the elicitation of T-cell mediated immune responses [43,44]. The total IgG antibody titers induced by Cat, Fn and Fn1 proteins were significantly higher than those elicited by the positive control of rsScpA193 protein, indicating that these segmented proteins possessed much stronger immunogenicity than rsScpA193 protein. Moreover, PA and Fn2 elicited comparable levels of total IgG antibody titers compared to those induced by rsScpA193, suggesting that both segment proteins were immunogenically similar to rsScpA193 protein.

The subtypes of antigen-specific IgG antibodies induced by each protein were also evaluated. Likewise, except for Fn3 protein, all other recombinant proteins provoked high levels of IgG1, IgG2a, and IgG2b antibodies (Figure 4D–F) and a lower IgG3 antibody response (Figure 4G). Both Cat and Fn proteins stimulated robust IgG1 antibody levels that were significantly higher than that induced by rsScpA193, whereas PA, Fn1 and Fn2 proteins exhibited much weaker abilities in stimulating anti-IgG1 responses. Moreover, the levels of IgG2a and IgG2b antibodies induced by all proteins were comparable. Taken together, these observations disclosed that most of the segment proteins showed strong immunogenicity, especially for Cat, Fn and Fn1 proteins, suggesting that these truncated proteins might be promising target immunogens for subunit protein vaccine development.

**Figure 5:**
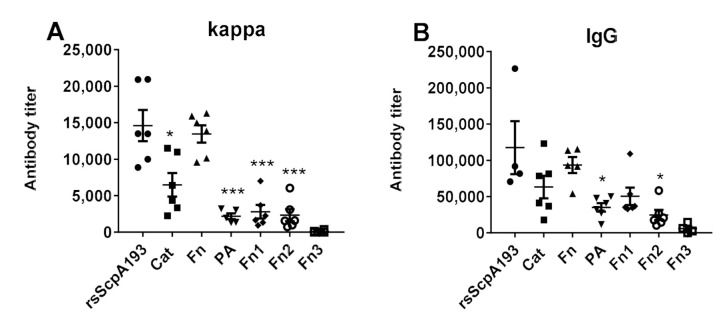
ELISA results of (**A**) total (anti-kappa) and (**B**) total IgG antibody titers in the pooled day 35 antisera detected with rsScpA protein as the capture antigen. Each dot represents the result of an individual mouse immunized with rsScpA193 (●), Cat (■), Fn (▲), PA (▼), Fn1 (♦), Fn2 (○), and Fn3 (□), respectively; the black bars show the mean ± SEM values. *: Significantly different (*p* < 0.05); ***: extremely significantly different (*p* < 0.001) from the data of antiserum rsScpA193.

Furthermore, the cross-reactions of the day 35 antisera of each segment protein toward the wild-type rsScpA were examined by ELISA to detect rsScpA-specific total (anti-kappa) antibodies and total IgG antibodies using rsScpA protein as the coating antigen. The results were outlined in Figure 5. The pooled antisera of each protein exhibited different binding results against rsScpA. Both total anti-kappa and IgG antibodies provoked by Fn protein exhibited much higher binding to rsScpA protein, which were comparable to those of rsScpA193 control. Both Cat and Fn1 proteins-induced total anti-kappa antibodies showed a relatively weaker binding to rsScpA than rsScpA193, whereas they produced considerable IgG antibodies that could significantly cross-react with rsScpA protein. Moreover, the antisera of PA and Fn2 showed much weaker cross-reactivity with rsScpA protein. These results indicated that antibodies elicited by recombinant Cat, Fn and Fn1 proteins might be recognizable by natural ScpA protein exposed on GAS cell surface.

### 3.3. Antiserum Binding to GAS and GBS Bacterium

**Figure 6:**
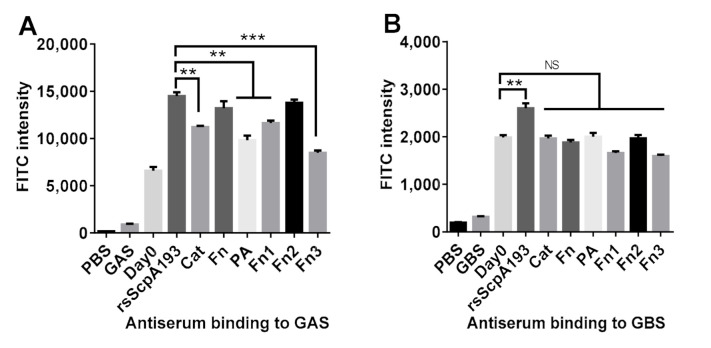
FCM assay of the binding capacities between (**A**) GAS and (**B**) GBS cells and the antisera (day 56, 1/50 dilution) pooled from mice immunized with segmented domains of ScpA and normal (day 0) mouse sera as negative controls. PBS: phosphate-buffered saline; GAS: *S. pyogenes* J17A4 (ATCC 12385); GBS: *S. agalactiae* (ATCC BAA-1138). NS: Not statistically significant; **: Very significantly different (*p* < 0.01), ***: Extremely significantly different (*p* < 0.001).

To further verify that the antisera of each protein would really recognize the ScpA protein on GAS cells, the in vitro antiserum-cell binding assay was investigated using flow cytometry (FCM). In this study, the day 56 antisera of each protein were used to disclose the perdurability and effectiveness of the elicited IgG antibodies. Therefore, after incubation of prefixed *S. pyogenes* J17A4 (ATCC 12385) cells with each pooled antiserum collected on day 0 (as negative control) and day 56, FITC-labeled goat anti-mouse IgG antibody was added to stain the antiserum-treated GAS cells. The binding capacities of each antiserum toward GAS cells were then analyzed by FCM, and the results were depicted in Figure 6A. As compared to GAS cells treated with day 0 antisera, GAS cells treated with the day 56 antisera all displayed statistically significant increases in fluorescence intensities, which indicated that immunization of these segment proteins in mice could produce the functional antibodies that recognized and bond to ScpA on GAS cell. The antiserum of Fn protein displayed the comparative binding activity to GAS cells as that of rsScpA193 serum. These results were consistent with those observed in the antibody titers assay (Figure 4) and the cross-reactivity analysis (Figure 5). Although Cat and Fn1 provoked significantly high titers of IgG antibodies (Figure 4B) and better cross-reactivity toward rsScpA protein (Figure 5B), the binding activities of their antisera to GAS cells were significantly weaker as compared with that of antiserum rsScpA193. Most interestingly, although Fn2 protein exhibited weaker immunogenicity (Figure 4C) in the antibody titers assay, it showed much stronger binding activity than other segmented proteins (except for Fn protein). This observed result was also comparable to that of rsScpA193 protein in statistics (Figure 6A). Furthermore, the antisera of PA and Fn3 protein had moderate binding activities that were comparable to those of Cat and Fn1 proteins. Taken together, these results revealed that the antisera of Fn and Fn2 proteins possessed the more functional IgG antibodies that could strongly bind to GAS cells. Accordingly, both Fn and Fn2 proteins together with rsScpA193 protein were identified as the more promising antigen candidates for protein-based GAS vaccine development.

It has been disclosed that C5a peptidases from different groups of streptococci are highly homologous in gene sequences (>95%) and thus antigenically conserved [45,46]. Therefore, we further evaluated the binding ability of each antiserum toward GBS. In this regard, prefixed *S. agalactiae* (ATCC BAA-1138) cells were incubated with the pooled antisera from each mouse group immunized with segment proteins, and the binding abilities of each antiserum were measured using the same FCM protocols described above. Remarkably, as shown in Figure 6B, only the antisera of rsScpA193 protein exhibited the significant binding activity towards GBS cells, whereas the antisera of all other segment proteins did not show any binding activities to GBS cells. This result indicated that only rsScpA193 protein might possess similar antigenic epitopes and spatial structure as ScpB of GBS [45,46,47]. Collectively, our study disclosed that rsScpA193 protein could elicited effective antibodies to recognize and bind to both GAS and GBS cells, indicating that it might provide the broad spectrum of immunological protection against GAS and GBS infections.

### 3.4. Preparation and Immunological Evaluation of GAC Trisaccharide-Protein Conjugates with Fn, Fn2 and rsScpA193 as Carrier Proteins

**Figure 7:**
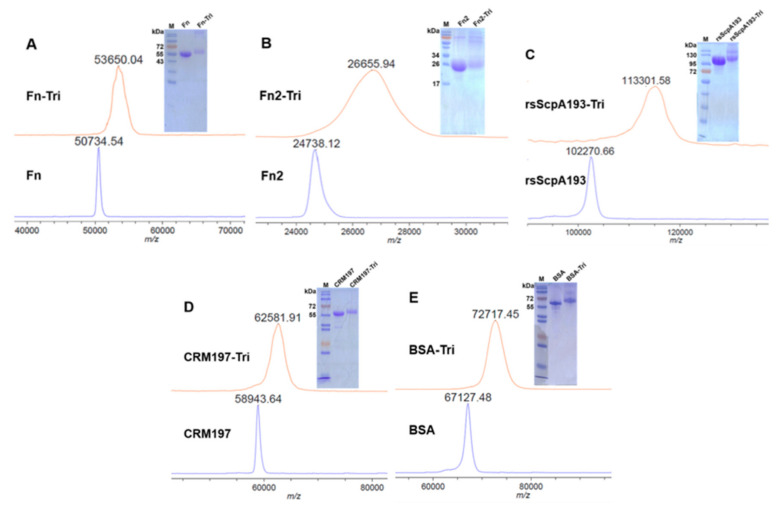
MALDI-TOF MS and SDS-PAGE results for (**A**) Fn and Fn-trisaccharide conjugate (Fn-Tri), (**B**) Fn2 and Fn2-trisaccharide conjugate (Fn2-Tri), (**C**) rsScpA193 and rsScpA193-trisaccharide conjugate (rsScpA-Tri), (**D**) CRM197 and CRM197-trisaccharide conjugate (CRM197-Tri) and (**E**) BSA and BSA-trisaccharide (BSA-Tri) conjugate.

In our previous study, we proved that ScpA193 protein could serve as a potential carrier protein for glycoconjugate vaccine development and its conjugation with GAC oligosaccharide haptens could help converting nonimmunogenic oligosaccharides into T-cell dependent antigens [14,15,39]. Given the strong immunogenic properties of Fn and Fn2 proteins and better binding abilities of their antisera to GAS bacterium as observed above, we further probed their potential as carrier proteins of glycoconjugate vaccines using GAC trisaccharide as the model antigen (Figure S1). Accordingly, Fn, Fn2 and rsScpA193 and CRM197 proteins were conjugated with GAC trisaccharide using the reactive di(*N*-succinimidyl)glutarate (DSG) as the conjugate linker according to the previously reported method (Figure S1) [14]. The glycoconjugates of Fn-Tri (Fn-trisaccharide conjugate) and Fn2-Tri (Fn2-trisaccharide conjugate) were prepared to examine the characteristics of Fn and Fn2 as potential carrier proteins, whereas rsScpA-Tri (rsScpA193-trisaccharide conjugate) and CRM197-Tri (CRM197-trisacchairde conjugate) were prepared and evaluated as positive controls. In addition, BSA-Tri (BSA-trisaccharide conjugate) was prepared as the coating antigen for detection of GAC oligosaccharide-specific antibodies in ELISA assay. The carbohydrate loading levels of GAC trisaccharide attached to the resultant proteins were determined by MALDI-TOF MS study and SDS-PAGE analysis (Figure 7) and the results depicted in Table 1 indicated that the trisaccharide loadings of these glycoconjugates were all in the desired range of 5~10% for glycoconjugate vaccines.

**Figure 8:**
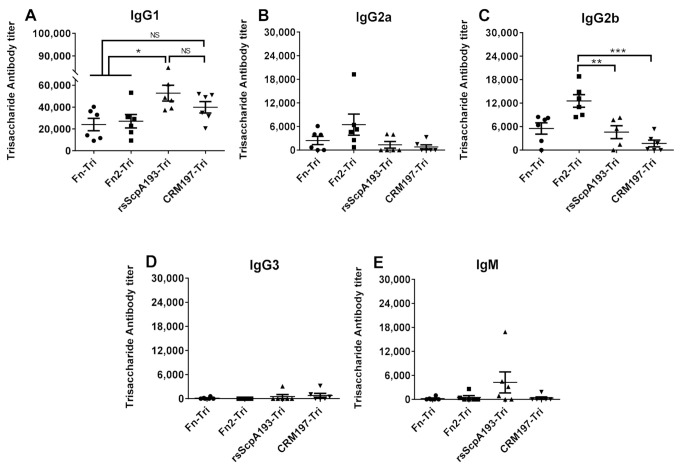
ELISA results showing the (**A**) IgG1, (**B**) IgG2a, (**C**) IgG2b, (**D**) IgG3 and (**E**) IgM antibody titers in the day 35 antisera of mice immunized with Fn-Tri (●), Fn2-Tri (■), rsScpA193-Tri (▲), and CRM197-Tri (▼), respectively, using BSA-Tri as the capture antigen. Each dot represents the result of an individual mouse, and the black bars show the mean ± SEM values. NS: Not statistically significant; *: significantly different (*p* < 0.05) **: very significantly different (*p* < 0.01); ***: extremely significantly different (*p* < 0.001).

Immunization of mice with each conjugate (Fn-Tri, Fn2-Tri, rsScpA193-Tri and CRM197-Tri) that was completely emulsified either with FCA or FIA following the same protocols described above was performed via subcutaneous injection to a group of six female BALB/c mice (3 μg of carbohydrate antigen per mouse) on days 1, 15, 22 and 29, respectively. In the meantime, the antisera were prepared via collection of blood samples from each mouse on day 0 before initial immunization (as blank control) and on day 35 after boosting immunizations. Thereafter, the obtained antisera were analyzed by ELISA to detect GAC trisaccharide-specific IgM, IgG1, IgG2a, IgG2b and IgG3 antibodies using BSA-trisaccharide conjugate as the coating antigen. The ELISA results were shown in Figure 8.

As frequently observed in other neoglycoprotein vaccines [48,49], all tested protein conjugates provoked high titers of trisaccharide-specific IgG1, IgG2a and IgG2b antibodies but low levels of IgG3 and IgM antibodies (Figure 8). This observation revealed the stimulation of T cell-mediated immune responses and the switch of antibody class [43,44]. Fn-Tri and Fn2-Tri conjugates elicited comparable IgG1 antibody titers (Fn: 24,058 ± 5656; Fn2: 27,080 ± 6150) as the positive control of CRM197-Tri (39,809 ± 5241), whereas the antibody titers were significantly lower than those induced by rsScpA193-Tri (52,766 ± 7276). These results indicated that, in comparison with rsScpA193 protein and commonly used CRM197 protein as carriers, Fn and Fn2 proteins as carriers possessed the comparative capability on conversion of nonimmunogenic trisaccharide into T-cell dependent antigen after their covalent conjugation. Furthermore, Fn2-Tri also elicited stronger IgG2a and IgG2b antibody responses (Figure 7B,C) than those of rsScpA193 and CRM197 conjugates. It has been well documented that IgG2b antibody had better binding to Fc receptors [50] and exhibited better opsonophagocytosis than IgG1 antibody [51,52]; thus, it has been characterized as the most dominant antibodies for activation of the immune system [52]. Our results suggested that Fn2 protein might be a promising carrier protein for the development of glycoconjugate vaccines.

## 4. Conclusions

In this study, we designed and constructed six segment proteins of ScpA, including Fn, PA, Fn1, Fn2, Fn3 and nontoxic Cat and the nearly full-length rsScpA193. Immunological study in mice demonstrated that, except for Fn3 protein, all other recombinant proteins could provoke robust T-cell mediated immune responses, which is crucial for prophylactic vaccines. Moreover, the IgG antibody titers induced by Cat, Fn, Fn1 and Fn2 were significantly higher than or comparable to those of rsScpA193 protein, and also showed better cross-reactivity with the wild-type rsScpA protein. Furthermore, the in vitro binding assay disclosed that the antisera of Cat, Fn, Fn1 and Fn2 exhibited significant binding activities toward GAS cell. More importantly, the antisera of Fn and Fn2 possessed better binding toward GAS cells as compared to that of rsScpA193. These finding suggested that Cat, Fn, Fn1 and Fn2 proteins, especially Fn and Fn2, could be promising anti-GAS subunit protein vaccine candidates. In addition, we also examined the binding ability of the antisera induced by all recombinant proteins to GBS cells, and the results disclosed that only the antisera of rsScpA193 protein exhibited significant binding activity. This result indicated that the relative full-length mutant rsScpA193 protein might possess similar antigenic epitopes and spatial structure as ScpB protein, a homology protein presented in GBS. Thus, together with the results in our previous reports [39], rsScpA193 could be identified as a promising antigen for the development of protein vaccine against both GAS and GBS infections.

To verify the potential of Fn and Fn2 as carrier proteins of glycoconjugate vaccines, we selected a GAC trisaccharide hapten as the model antigen, and conjugated it with Fn, Fn1, rsScpA193 and CRM197 protein, respectively, to generate oligosaccharide-protein conjugates. Immunological studies of these conjugates disclosed that all conjugates elicited strong GAC trisaccharide-specific T-cell dependent immune responses, which undoubtedly indicated that Fn and Fn2 as carrier proteins could convert immunologically inactive trisaccharide into a T-cell dependent immunogenic antigen. Although the antibody titers induced by Fn-Tri and Fn2-Tri conjugates were significantly weaker than that of rsScpA193-Tri conjugate, they were comparable to that of CRM197-Tri conjugate. In addition, both Fn-Tri and Fn2-Tri conjugates could also provoke high levels of IgG2a and IgG2b antibody responses. The IgG2b antibody response elicited by Fn2-Tri conjugate was significantly stronger than those of rsScpA193-Tri and CRM197-Tri conjugate. As IgG2b antibody was important and useful for stimulation of the immune system, our results indicated Fn and Fn2 proteins, especially Fn2 protein, had great potential application as carrier molecules in glycoconjugate vaccine development.

In conclusion, the results of our preliminary immunological studies on a series of subunit proteins of ScpA (i.e., Fn, Fn2, rsScpA193) and their trisaccharide conjugates have revealed their great potential as immunogens in development of novel ScpA subunit protein-based anti-GAS vaccine and as carrier proteins for the formulation of functional carbohydrate-based conjugate vaccines. Further investigation on immunological properties of these promising candidates including antibody binding activities between different serotype species, in vitro and in vivo antibacterial studies are currently underway in our laboratory.

## Figures and Tables

**Table 1:** Carbohydrate Loading of Glycoconjugates.

Glycoconjugate	Fn-Tri	Fn2-Tri	rsScpA193-Tri	CRM197-Tri	BSA-Tri
Oligosaccharide chains /carrier protein (m)	4.3	2.8	16.3	5.5	8.3
Carbohydrate loading (%)	5.4	7.2	9.7	5.8	7.7

## Data Availability

Data available upon request.

## Supplementary Materials

The following are available online at https://www.mdpi.com/2076-393X/9/2/139/s1, Table S1: The primers designed for each segmented domain of ScpA; Figure S1: Conjugation of GAC trisaccharide with different carrier proteins.
