# Supplementary material for: Immunogenicity Assessment of Different Segments and Domains of Group a Streptococcal C5a Peptidase and Their Application Potential as Carrier Protein for Glycoconjugate Vaccine Development
Source: Vaccines (Basel). 2021 Feb 9;9(2):139. doi: 10.3390/vaccines9020139 (PMC7915350; doi:10.3390/vaccines9020139)
Supplement: Supplementary file 1 [file vaccines-09-00139-s001.pdf]

vaccines MDPI

Table 1. The primers designed for each segmented domain of ScpA.

Entry	protein	vector		Primers 5'→3'
1	rsScpA	pGEX-6p-3	forward	GGGATCCACCTCAAAAGCGACTA
			reverse	GCTCGAGTCAAGAGTGGCCCTCC
2	rsScpA193	pGEX-6p-3	forward	GGGATCCACCTCAAAAGCGACTA
			reverse	GCTCGAGTCAAGAGTGGCCCTCC
3	Cat	pET-21b	forward	GCATATGACCTCAAAAGCGACTA
			reverse	GCTCGAGTGCTGCTGAAGCTTTT
4	Fn	pGEX-6p-3	forward	CGGATCCACGATGTATGTGACAG
			reverse	GCTCGAGTCAAGAGTGGCCCTCC
5	PA	pGEX-6p-3	forward	GGGATCCCCAGA TAAAC AGCTCA
			reverse	GCTCGAGTCAGGTGTCACTTGCTGT
6	Fn1	pGEX-6p-3	forward	GGGATCCACGATGTATGTGACAG
			reverse	GCTCGAGTCAATCACCTCGGAAA
7	Fn2	pGEX-6p-3	forward	CGGATCCTTTGGCAATCTGTCAG
			reverse	GCTCGAGTCAATTGTCTACAATCA
8	Fn3	pGEX-6p-3	forward	CGGATCCACGACACCTGAAGTCG
			reverse	GCTCGAGTCAAGAGTGGCCCTCC

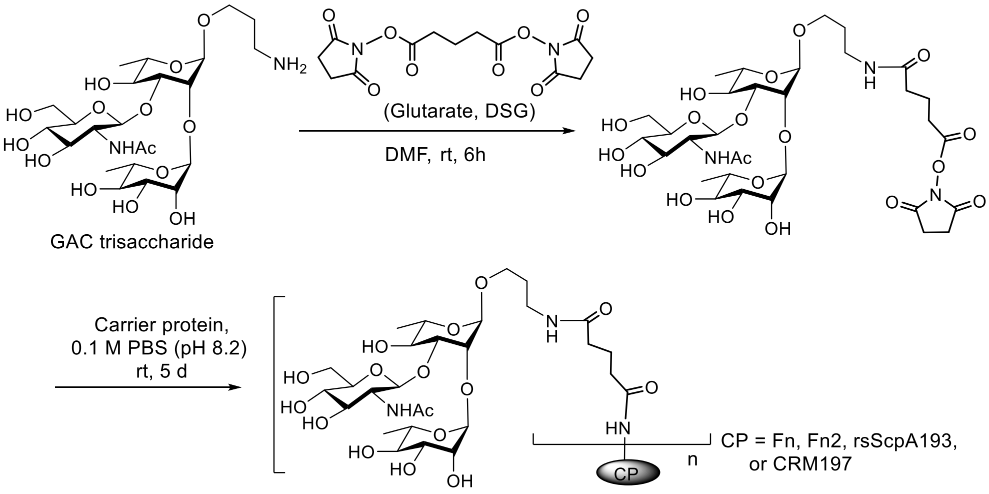

Figure 1. Conjugation of GAC trisaccharide with different carrier protein via bifunctional glutaryl linker.
